# Effect of a Leucine/Pyridoxine Nutraceutical on Caloric Intake and Body Composition of Obese Dogs Losing Weight

**DOI:** 10.3389/fvets.2020.00555

**Published:** 2020-08-25

**Authors:** Maryanne Murphy, Joseph W. Bartges, Michael B. Zemel, Claudia A. Kirk, Angela Witzel-Rollins

**Affiliations:** ^1^Department of Small Animal Clinical Sciences, University of Tennessee College of Veterinary Medicine, Knoxville, TN, United States; ^2^NuSirt Biopharma, Research & Development, Knoxville, TN, United States

**Keywords:** obesity, dog, canine, leucine, pyridoxine, nutraceutical

## Abstract

The aim of this 29-week randomized, positively and negatively controlled study was to investigate whether a nutraceutical containing 1 g leucine and 13 mg pyridoxine can enhance weight loss while maintaining lean muscle mass in obese dogs. Twenty-four healthy, 2-year-old beagles were initially divided into obesification (*n* = 18) or ideal body weight groups (*n* = 6). After obesification, the 18 dogs were divided into three weight loss groups and fed one of the following over 12 weeks: nutraceutical with canned adult diet (CAD; ObN), placebo with CAD (ObP), or a canned therapeutic weight loss diet (WLD). Dogs in the ideal body weight (IBW) group were fed maintenance calorie requirements with CAD over 12 weeks. Based on MANOVA, ObN and WLD lost similar amounts of total weight (3.6 ± 0.9 vs. 4.4 ± 1.1 kg, respectively) and fat mass (3.1 ± 0.6 vs. 3.9 ± 0.8 kg, respectively) after 12 weeks of treatment, and more than ObP (1.1 ± 1.2 kg weight; 0.9 ± 1.0 kg fat; *p* < 0.0001). These data show the nutraceutical is a promising option for successful weight loss in dogs. Maintenance levels of CAD were able to induce weight loss without risk of hypo- or anorexia, or the need to switch diets or restrict energy intake.

## Introduction

Global estimates published within the last 10 years indicate 41–65% of adult dogs are either overweight or obese (1–5). The greatest prevalence is from dogs 6–10 years old, neutered, and represented most commonly by specific breeds [e.g., Beagle, Labrador Retriever, and Rottweiler; (6)]. Lean Labrador Retrievers have been found to live 1.8 years longer and require long-term treatment for osteoarthritis 3 years later than their overweight sex- and weight-matched peers (7). Overweight and obese dogs also have reduced health-related quality of life, providing strong support for veterinary recommendations for weight loss to improve health and longevity in the dog (8–10).

Diet manipulations to decrease caloric intake have long been the treatment of choice for canine obesity, but adherence and long-term weight maintenance success has proven difficult to achieve (11–18). Most plans involve feeding specially designed therapeutic weight loss diets, typically providing an increased nutrient to calorie ratio, increased fiber and protein content, and reduced kcal/kg food content (15, 19). In cases where switching the diet is not desirable, alternative methods of calorie restriction are required.

In people, a nutraceutical combining 2.25 g leucine and 30 mg pyridoxine has shown promise as an effective aid to manage obesity (20, 21). The premise involves the synergistic ability of the two nutrients to encourage lipolysis, while maintaining lean tissue mass. Leucine is an essential ketogenic branched chain amino acid (BCAA), with highest concentrations (g leucine/100 g food item) in eggs, soy-derived products, and dairy products (22). Leucine has been shown to modulate fat oxidation and energy partitioning between adipose tissue and skeletal muscle. The cross-talk that occurs causes diminished adipocyte lipid storage, increased net fat oxidation, and decreased overall adiposity (21, 23–28). Pyridoxine (vitamin B6) is a cofactor for various macronutrient metabolism enzymes in its active form, pyridoxal 5′ phosphate (PLP). It tends to be highest in fortified foods, but naturally occurring sources of pyridoxine with high concentrations (mg pyridoxine/100 g food item) are pistachios, wheat and rice bran, sunflower seeds, and various spices (22). PLP from pyridoxine supplementation has been shown to attenuate calcium signaling and decrease net lipid storage (20, 21).

The central aim of the study was to determine if a leucine/pyridoxine supplement would enhance weight loss and maintain lean tissue mass in obese dogs losing weight compared to a negative placebo control and a positive weight reduction diet control. We hypothesized the nutraceutical would produce weight loss results similar to the therapeutic weight loss diet, and both diets would induce increased body fat loss with maintenance of lean muscle mass compared to placebo. Dogs maintaining their weight were expected to have consistent results at every time point and were included as a negative control.

## Materials and Methods

### Animals

Twenty-four healthy, 2-year-old male castrated Beagles were assigned to four groups using a random number table. Individual groups were maintained in the same room, and all dogs were individually housed in runs within the laboratory animal facilities of the University of Tennessee College of Veterinary Medicine. Exercise was provided for 30 min two times per day for the duration of the trial. Dogs were deemed healthy prior to inclusion based on physical examination, complete blood counts, plasma biochemical analysis, and urinalysis. The study protocol was reviewed and approved by The University of Tennessee Institutional Animal Care and Use Committee.

**Figure 1:**
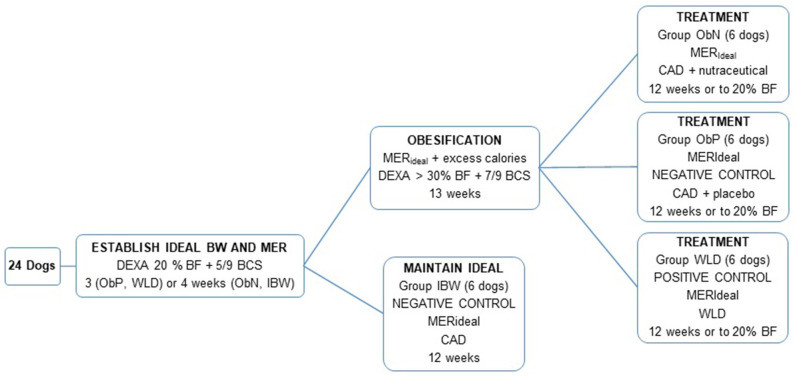
Group assignments. BW, body weight; MER, maintenance energy requirement; DEXA, dual energy X-ray absorptiometry; % BF, percent body fat; BCS, body condition score; IBW, ideal body weight; ObN, obese nutraceutical; ObP, obese placebo; WLD, weight loss diet; CAD, canned adult diet.

Dogs were fed based on group assignments and calculated maintenance energy requirements as described below (see Figure 1). The groups were assigned as followed: obese nutraceutical group, ObN; obese placebo group, ObP; obese therapeutic weight loss diet group, WLD; ideal body weight group, IBW; and each group contained 6 dogs.

### Pre-study Period

Resting energy requirement (RER; 70 × BW_kg_^0.75^) was calculated for each dog based on calculated ideal body weight. Ideal body weight [(current weight x current % lean)/ideal % lean] was calculated using the estimated percent body fat (% BF) relating to each dog's current body condition score (BCS) based on a 9-point scale (29, 30). Current % lean was determined by subtracting the current % BF from 100, and 80% was used as the ideal lean value. Maintenance energy requirement (MER; RER × lifestage factor) was calculated using a lifestage factor of 1.6, corresponding to the recommendation for a castrated adult dog (31).

Dogs were fed a canned adult diet (CAD; Table 1) at MER divided into two daily meals for three (ObP, WLD) or 4 weeks (ObN, IBW). A 4-week period was planned for all groups, but research facility limitations necessitated a slightly shorter timeline for half of the dogs. Using a random number table, ObN and IBW were assigned to the 4-week period, while ObP and WLD were fed for 3 weeks. Dogs had access to food for 45 min at each feeding, and food intake was recorded by measuring residual food weight. Body weight and BCS were recorded three times a week, and feeding amount was adjusted to establish the individual's ideal MER (MER_ideal_) based on achieving or maintaining lean body weight and BCS.

**Table 1:** Diet nutrient profiles.

**Nutrients (per 1000 kcal)**	**CAD diet**	**WLD diet**	**Kitten diet**
Protein (g)	56	85	85
Fat (g)	38	29	57
Carbohydrate (g)	129	131	61
Crude fiber (g)	2	71	3
Leucine (g)	5.0	6.0	9.2
Isoleucine (g)	2.0	2.7	3.5
Valine (g)	2.6	4.1	3.9
Pyridoxine (mg)	2.4	3.3	2.4
Metabolizable energy (kcal/kg)	1021	733	4196

*Information provided by manufacturer 2012 product guide average nutrient analysis. Values not listed in product guide were obtained directly from manufacturer*.

*CAD, canned adult diet (Science Diet® Adult Canned Gourmet Beef Entrée, Hill's Pet Nutrition, Inc., Topeka, KS, USA); WLD, weight loss diet (Hill's Prescription Diet® r/d®, Hill's Pet Nutrition, Inc., Topeka, KS, USA); kitten diet (Science Diet® Kitten Healthy Development, Hill's Pet Nutrition, Inc., Topeka, KS, USA)*.

### Obesification

Three groups (ObN, ObP, and WLD) participated in a 13-week obesification period. Established MER_ideal_ was continued for ObN, ObP, and WLD dogs. Body weight and BCS determination was decreased to twice weekly. In order to induce an overweight or obese state (%BF >30) excess calories were provided from canola oil, vegetable oil, and extruded kitten food. Nutrient profile of the kitten diet is shown in Table 1. Oils were provided as 10 g each, with both meals based on a previously published protocol (32). Due to intermittent soft stools and decreased appetite noted in some dogs, oil dose was decreased by 50% after 5 weeks. Kitten food was given in ¼ cup increments and increased as needed to encourage weight and % BF gain over the final 4 weeks. Dogs that lost or maintained a consistent weight over 1 week during obesification had ¼ can CAD added to their daily food ration. Cans were 370 g and each ¼ increment added ~92.5 g.

The forth group (IBW) maintained their weight on CAD at established MER_ideal_ as a negative control for 12 weeks in conjunction with the obesification period. Body weight and BCS were determined once a week. Feeding amount was adjusted in ¼ can increments as needed to maintain ideal body weight and condition. After the 12-week period, IBW dogs had completed the requirements for the experiment and were not used during the treatment period.

### Treatment

Excess calories (oil and kitten food) were removed, and the three groups undergoing obesification were maintained on MER_ideal_ from CAD (ObN and ObP) or therapeutic weight loss diet (WLD; Table 1). All feeding amounts were decreased to a maximum of 3 cans CAD (1133.3 kcal) or 4.5 cans WLD (1154.5 kcal) per day. The average body weight of all eighteen dogs was 16.0 kg (RER 560 kcal; RER × 1.6 = 896 kcal, MER), so this caloric level limited the dogs to ~20% above calculated MER (~2 × RER). ObN dogs received two nutraceutical capsules (1 g leucine + 13 mg pyridoxine total dose), and ObP dogs received two placebo capsules (corn starch) in a CAD meatball immediately before getting access to their morning meal. The weight (g) of the capsule meatball was recorded as part of the morning meal weight. The combined diet and nutrient content levels based on average caloric intake at study completion across the treatment groups are shown in Table 2.

**Table 2:** Diet and treatment nutrient profiles based on average calorie consumption.

Nutrients (per 864 kcal^*^)	**Nutraceutical and CAD (ObN)**	**Placebo and CAD (ObP)**	**Weight loss diet (WLD)**
Leucine (g)	5.3	4.3	5.2
Isoleucine (g)	1.7	1.7	2.4
Valine (g)	2.2	2.2	3.5
Pyridoxine (mg)	15.1	2.1	2.9

* *Calorie level based on average consumption at study completion for the 15 dogs from groups ObN, ObP, and WLD that were used in final data analysis*.

*Values based on average nutrient analysis information obtained directly from manufacturer*.

*CAD, canned adult diet (Science Diet® Adult Canned Gourmet Beef Entrée, Hill's Pet Nutrition, Inc., Topeka, KS, USA); WLD, weight loss diet (Hill's Prescription Diet® r/d®, Hill's Pet Nutrition, Inc., Topeka, KS, USA)*.

*ObN, obese nutraceutical group; ObP, obese placebo group; WLD, weight loss diet group*.

During treatment, body weight and BCS determination was decreased to once a week. Treatment was continued until the dog reached 20% BF or up to 12 weeks. For dogs reaching ideal body composition before 12 weeks, the body fat level as estimated by BCS was confirmed using dual energy X-ray absorptiometry (DEXA) prior to treatment termination.

### Caloric Consumption

To avoid confounding changes in food consumption due to fasting prior to DEXA, caloric intake calculations were based on average food consumption over 4 days at the end of obesification (baseline) and 1, 4, 8, and 12 weeks into treatment. The fifth day of food recording was dropped to account for morning meal loss and poor afternoon meal consumption post-anesthesia on DEXA days. For the duration of this report, all calories offered and consumed are based on metabolic body weight (kcal/BW_kg_^0.75^). In addition, calories offered and consumed during obesification are credited only to CAD to allow for direct comparison of CAD consumption during all phases of the study.

### Body Composition

Body composition was determined using HOLOGIC® fan-beam QDR 4500A DEXA (Hologic, Inc., Bedford, MA) at the end of obesification and before starting treatment, serving as the baseline measurement. Dogs were fed their standard evening meal the day prior and then fasted the morning of analysis. An intravenous or intramuscular injection of butorphanol (0.2 mg/kg) and acepromazine (0.05 mg/kg) was given as premedication 10–20 min prior to induction. Propofol (2–8 mg/kg, to effect) was given intravenously, and dogs were immediately placed in ventral recumbency on the DEXA table. Scans were performed using the whole body fan beam setting and analyzed using APEX System Software version 2.3 (Hologic, Inc., Bedford, MA) by the same operator. DEXA was performed again under general anesthesia 1, 4, 8, and 12 weeks into treatment. Additional scans were performed as needed during weight loss to confirm study termination in those dogs reaching ideal body condition prior to 12 weeks.

### Statistical Analysis

Based on a power calculation, six dogs per group were considered sufficient to demonstrate significant differences. The calculation utilized assumptions of a difference in mean body fat between treated (ObN) and untreated (ObP) dogs of 15% (increase in body fat from 15 to 25% in treated and 15–40% in untreated dogs), a within standard deviation of 25% in body fat, an alpha of 0.05, and a beta of 0.9. Data was analyzed using MANOVA for repeated measures with SAS® version 9.4 (SAS Institute, Inc., Cary, NC). Rank transformations were applied on variables that exhibited violation of normality and equal variance model assumptions. Multiple comparisons within group, time, and their interaction effects were corrected with Tukey's HSD. *P* < 0.05 were reported as statistically significant.

## Results

Twenty-four dogs started the study and 21 dogs were included in final data analysis. Three dogs (one from each obesification group) did not reach the required 30% BF threshold during obesification and were dropped from analysis. One dog from WLD reached ideal body weight (18.2%BF) at week 8 and ended study participation at that point.

### Pre-study Period

Average starting weight and BCS during the MER_ideal_ establishment phase for all dogs was 11.7 ± 1.0 kg and 4.6 ± 0.7, respectively. Starting weights were 0.3 ± 0.74 kg above estimated ideal weight (~20 %BF) for ObN dogs and 0.2 ± 0.31 kg below for IBW dogs. All six ObN dogs gained weight while consuming calculated MER_ideal_ during the first week, and all six IBW dogs lost weight. One week later, when the other two groups were started, groups were both above (ObN, 0.6 ± 0.75 kg; WLD, 0.1 ± 0.30 kg) and below (ObP, 0.2 ± 0.52 kg; IBW, 0.8 ± 0.38 kg) their estimated ideal weights. Reassessment 1.5 weeks later (1.5 or 2.5 weeks into MER_ideal_ establishment phase) revealed ObN dogs were 0.2 ± 0.57 kg above and all other groups were below (ObP, 0.8 ± 0.42 kg; WLD, 0.5 ± 0.22 kg; IBW, 0.6 ± 0.44 kg) their estimated ideal weights. Feeding amounts were increased for dogs not meeting weight gain targets (1/6 ObN, 3/6 ObP, 3/6 WLD, 6/6 IBW), and they were decreased for those above target weight (3/6 ObN, 1/6 ObP, 2/6 WLD) to compensate for these weight changes.

During obesification, CAD amounts offered were increased in dogs that lost or maintained a consistent weight. By the end of obesification (baseline), three ObN dogs were being fed at or above calculated MER_ideal_ (1.0–2.2 × MER_ideal_, 1.6–3.5 × RER_ideal_), and three dogs were eating below calculated MER_ideal_ (0.5–0.9 × MER_ideal_, 0.9–1.5 × RER_ideal_). This is in comparison to the other obesification groups, in which 10 dogs were being fed at/above (ObP, 1.2–2.0 × MER_ideal_, 1.8–3.3 × RER_ideal_; WLD, 1.0–2.2 × MER_ideal_, 1.6–3.5 × RER_ideal_) and two were fed below (WLD, 0.9 × MER_ideal_, 1.4 × RER_ideal_) calculated MER_ideal_.

### Treatment Scale Weight and BCS

There were no differences in body weight (BW) and BCS at baseline or 1 week into treatment within or between obesified groups. At the end of treatment, BW and BCS for group ObP were not different from baseline or 1 week values. In contrast, compared to baseline and 1 week, BW and BCS for group ObN and WLD were significantly different from their respective ending values, but were not different from each other. Group ObP end of treatment BW was different from ObN and WLD (*p* < 0.0001), but mean BCS did not differ for group ObP vs. ObN, while WLD differed from both (*p* < 0.0352; Table 3). Based on change from baseline, ObN and WLD lost similar levels of BW after 12 weeks of treatment and more than ObP (*p* < 0.0001; Table 4).

**Table 3:** Body weight, BCS, and caloric consumption by obesification group and time point (study week) during weight loss.

**Group**	**Baseline**	**1 week**	**4 weeks**	**8 weeks**	**12 weeks^1^**
**Body weight (kg)**					
ObN	16.4 ± 0.7^b^^,^^#^	16.0 ± 0.8^b^^,^^c^^,^^#^^,^^$^	15.0 ± 1.1^b^^,^^$^	13.7 ± 1.3^b^^,^^%^	12.9 ± 1.5^b^^,^^%^
ObP	17.2 ± 1.4^b^^,^^#^	17.0 ± 1.1^c^^,^^#^	16.8 ± 0.9^c^^,^^#^	15.9 ± 0.4^c^^,^^#^	16.0 ± 0.4^c^^,^^#^
WLD	16.3 ± 1.4^b^^,^^#^	15.3 ± 1.4^b^^,^^#^^,^^$^	14.7 ± 1.0^b^^,^^$^^,^^%^	13.9 ± 1.0^b^^,^^%^	12.4 ± 1.0^a^^,^^b^^,^^∧^
IBW	11.2 ± 0.8^a^^,^^#^	11.3 ± 0.7^a^^,^^#^	12.2 ± 0.7^a^^,^^#^^,^^$^	12.5 ± 0.8^a^^,^^$^	11.8 ± 0.9^a^^,^^#^^,^^$^
**BCS (9-point)**					
ObN	8.8 ± 0.4^b^^,^^#^	8.8 ± 0.4^b^^,^^#^	8.0 ± 0.7^b^^,^^#^	7.4 ± 0.9^b^^,^^#^	6.4 ± 0.9^b^^,^^$^
ObP	8.2 ± 0.8^b^^,^^#^	8.0 ± 1.0^b^^,^^#^	8.0 ± 0.7^b^^,^^#^	8.0 ± 1.4^b^^,^^#^	7.4 ± 0.9^b^^,^^#^
WLD	8.4 ± 1.3^b^^,^^#^	8.0 ± 1.2^b^^,^^#^^,^^$^	7.8 ± 1.1^b^^,^^#^^,^^$^	6.8 ± 1.3^b^^,^^$^	5.3 ± 1.0^a^^,^^%^
IBW	4.3 ± 0.8^a^^,^^#^	4.5 ± 0.5^a^^,^^#^	5.0 ± 0.0^a^^,^^#^	5.0 ± 0.0^a^^,^^#^	4.2 ± 0.4^a^^,^^#^
**Calories offered (kcal/BW**_**kg**_^**0.75**^**)**					
ObN	75.0 ± 17.2^a^^,^^#^	79.6 ± 16.1^a^^,^^#^^,^^$^	83.6 ± 14.9^a^^,^^#^^,^^$^	90.8 ± 11.3^a^^,^^#^^,^^$^	101.3 ± 12.7^a^^,^^$^
ObP	141.4 ± 41.2^b^^,^^#^	128.7 ± 19.2^b^^,^^#^	129.1 ± 18.6^b^^,^^#^	134.0 ± 16.6^b^^,^^c^^,^^#^	134.1 ± 17.3^b^^,^^#^
WLD	123.4 ± 58.9^b^^,^^#^	111.5 ± 40.2^b^^,^^#^	114.3 ± 38.4^b^^,^^#^	120.7 ± 38.5^b^^,^^#^	117.5 ± 31.1^b^^,^^#^
IBW	215.2 ± 42.6^c^^,^^#^	207.6 ± 40.2^c^^,^^#^	179.7 ± 32.7^c^^,^^#^^,^^$^	156.5 ± 31.6^c^^,^^$^^,^^%^	139.8 ± 38.9^b^^,^^%^
**Calories consumed (kcal/BW**_**kg**_^**0.75**^**)**					
ObN	63.1 ± 16.2^a^^,^^#^	79.6 ± 16.1^a^^,^^#^^,^^$^	83.6 ± 14.9^a^^,^^#^^,^^$^	90.8 ± 11.3^a^^,^^#^^,^^$^	101.3 ± 12.7^a^^,^^$^
ObP	88.6 ± 17.2^a^^,^^#^	120.3 ± 23.9^b^^,^^$^	128.1 ± 20.2^b^^,^^$^	134.0 ± 16.6^b^^,^^c^^,^^$^	134.1 ± 17.3^a^^,^^b^^,^^$^
WLD	87.2 ± 25.8^a^^,^^#^	87.7 ± 16.7^a^^,^^#^	113.7 ± 37.7^a^^,^^b^^,^^#^^,^^$^	120.7 ± 38.5^a^^,^^b^^,^^$^	112.7 ± 22.8^a^^,^^b^^,^^$^
IBW	215.1 ± 42.6^b^^,^^#^	207.6 ± 40.2^c^^,^^#^	179.7 ± 32.7^c^^,^^#^^,^^$^	156.5 ± 31.6^c^^,^^$^^,^^%^	139.7 ± 38.9^b^^,^^%^

*Numerical data presented as mean ± standard deviation*.

*ObN, obese nutraceutical group; ObP, obese placebo group; WLD, weight loss diet group; IBW, ideal body weight group*.

*Except where otherwise indicated, n = 5 for obesification groups (ObN, ObP, WLD) and n = 6 for group IBW*.

1 *WLD in this column determined from 4 dogs, because 1 dog reached ideal weight and ended treatment at 8 weeks*.

a,b,c *Within a column, values with different superscript letters differ significantly (p < 0.05)*.

#,$,%,∧ *Across columns, values with different superscript symbols differ significantly (p < 0.05)*.

**Table 4:** Body composition change from baseline by obesification group and time point during weight loss.

**Change from baseline**	**4 weeks**	**8 weeks**	**12 weeks^1^**
**Body weight (kg)**			
ObN	−1.4 ± 0.6^c^^,^^#^	−2.7 ± 0.8^c^^,^^$^	−3.6 ± 0.9^c^^,^^$^
ObP	−0.4 ± 0.6^a^^,^^#^	−1.3 ± 1.0^b^^,^^$^	−1.1 ± 1.2^b^^,^^$^
WLD	−1.6 ± 0.5^c^^,^^#^	−2.3 ± 1.0^c^^,^^#^	−4.4 ± 1.1^c^^,^^$^
IBW	−1.0 ± 0.6^b^^,^^#^^,^^$^	−1.3 ± 0.8^a^^,^^#^	−0.6 ± 0.9^a^^,^^$^
**Fat mass (kg)**			
ObN	−1.2 ± 0.3^b^^,^^c^^,^^#^	−2.2 ± 0.4^c^^,^^$^	−3.1 ± 0.6^c^^,^^$^
ObP	−0.4 ± 0.5^a^^,^^#^	−0.8 ± 0.7^a^^,^^#^	−0.9 ± 1.0^b^^,^^#^
WLD	−1.5 ± 0.4^c^^,^^#^	−2.7 ± 0.8^c^^,^^$^	−3.9 ± 0.8^c^^,^^$^
IBW	−0.7 ± 0.3^b^^,^^#^	−1.0 ± 0.6^b^^,^^#^	−0.7 ± 0.7^a^^,^^#^
**Body fat (%)**			
ObN	−3.4 ± 1.3^a^^,^^b^^,^^#^	−7.3 ± 1.7^c^^,^^$^	−12.1 ± 3.7^c^^,^^$^
ObP	−0.7 ± 1.0^a^^,^^#^	−2.4 ± 2.2^a^^,^^#^	−2.6 ± 3.2^a^^,^^#^
WLD	−5.2 ± 1.9^b^^,^^#^	−11.7 ± 3.2^c^^,^^$^	−17.0 ± 4.8^c^^,^^$^
IBW	−4.4 ± 2.3^c^^,^^#^	−6.6 ± 4.3^b^^,^^#^	−5.0 ± 4.7^b^^,^^#^
**Lean mass (kg)**			
ObN	−0.5 ± 0.3^b^^,^^#^	−0.6 ± 0.5^a^^,^^#^	−0.6 ± 0.3^b^^,^^#^
ObP	−0.3 ± 0.3^a^^,^^b^^,^^#^	−0.3 ± 0.2^a^^,^^#^	−0.4 ± 0.3^a^^,^^b^^,^^#^
WLD	−0.4 ± 0.6^b^^,^^#^	−0.4 ± 0.6^a^^,^^#^	−0.4 ± 0.5^a^^,^^b^^,^^#^
IBW	−0.1 ± 0.2^a^^,^^#^	−0.04 ± 0.3^a^^,^^#^	−0.05 ± 0.3^a^^,^^#^
**Lean (%)**			
ObN	3.0 ± 1.3^a^^,^^#^	6.7 ± 1.5^c^^,^^$^	11.3 ± 3.4^c^^,^^$^
ObP	0.5 ± 0.8^a^^,^^#^	2.1 ± 2.1^a^^,^^#^	2.4 ± 3.0^a^^,^^#^
WLD	4.8 ± 2.0^a^^,^^#^	10.7 ± 3.3^c^^,^^$^	16.2 ± 4.7^c^^,^^$^
IBW	4.2 ± 2.3^b^^,^^#^	6.3 ± 4.2^b^^,^^#^	4.9 ± 4.6^b^^,^^#^

*Numerical data presented as mean ± standard deviation*.

*ObN, obese nutraceutical group; ObP, obese placebo group; WLD, weight loss diet group; IBW, ideal body weight group*.

*Except where otherwise indicated, n = 5 for obesification groups (ObN, ObP, WLD) and n = 6 for group IBW*.

1 *WLD in this column determined from 4 dogs, because 1 dog reached ideal weight and ended treatment at 8 weeks*.

a,b,c *Within a column, values with different superscript letters differ significantly (p < 0.05)*.

#,$ *Across columns, values with different superscript symbols differ significantly (p < 0.05)*.

### Caloric Consumption

#### Calories Offered

There were no differences in amount of calories offered (kcal/BW_kg_^0.75^) within obesification groups at all time points, except 12 weeks (Table 3). At this final time point, group ObN was offered a greater amount of calories than was offered at baseline (*p* = 0.0116). Amount of calories offered to group ObN was significantly less than that offered to ObP and WLD, which were not different from each other, at all time points (*p* < 0.05). When the WLD dog that reached ideal body weight and ended study participation at 8 weeks is included in a comparison of baseline vs. end of study, amount of calories offered to WLD did not differ from ObN or ObP, which differed from each other (ObN, 101.3 ± 12.7 kcal/BW_kg_^0.75^; ObP, 134.1 ± 17.3 kcal/BW_kg_^0.75^; WLD, 127.9 ± 35.5 kcal/BW_kg_^0.75^; *p* = 0.0077).

#### Calories Consumed

Caloric intake between obese groups did not differ at obesification treatment baseline. One week into treatment, ObP consumed more calories than at baseline (*p* = 0.0046), and consumption remained unchanged for the duration. At week twelve, group ObN consumed more kcal/BW_kg_^0.75^ than at baseline (*p* = 0.001). WLD consumed more kcal/BW_kg_^0.75^ at weeks 8 and 12 than at baseline (*p* < 0.006). By the end of treatment, caloric consumption per kcal/BW_kg_^0.75^ was similar between all obesification groups.

### Body Composition

Body composition data for obesification groups are listed in Table 5.

**Table 5:** DEXA body composition by obesification group and time point (study week) during weight loss.

**Group**	**Baseline**	**1 week**	**4 weeks**	**8 weeks**	**12 weeks^1^**
**Fat mass (kg)**					
ObN	7.1 ± 0.5^b^^,^^#^	6.7 ± 0.5^b^^,^^#^	5.9 ± 0.6^b^^,^^c^^,^^#^^,^^$^	4.9 ± 0.7^b^^,^^c^^,^^$^^,^^%^	4.0 ± 1.0^b^^,^^%^
ObP	6.7 ± 1.0^b^^,^^#^	6.3 ± 0.7^b^^,^^#^	6.3 ± 0.7^c^^,^^#^	5.8 ± 0.7^c^^,^^#^	5.7 ± 0.8^c^^,^^#^
WLD	6.4 ± 1.4^b^^,^^#^	5.9 ± 1.4^b^^,^^#^^,^^$^	4.9 ± 1.1^b^^,^^$^^,^^%^	3.7 ± 0.9^a^^,^^b^^,^^$^^,^^%^^,^^∧^	3.1 ± 0.8^a^^,^^∧^
IBW	2.1 ± 0.5^a^^,^^#^	2.2 ± 0.4^a^^,^^#^	2.8 ± 0.4^a^^,^^#^^,^^$^	3.0 ± 0.4^a^^,^^$^	2.8 ± 0.3^a^^,^^#^^,^^$^
**Body fat (%)**					
ObN	42.9 ± 2.9^b^^,^^#^	42.9 ± 3.0^b^^,^^#^	39.5 ± 2.5^c^^,^^#^^,^^$^	35.6 ± 2.9^b^^,^^$^^,^^%^	30.8 ± 5.4^b^^,^^%^
ObP	38.7 ± 3.6^b^^,^^#^	37.3 ± 3.5^b^^,^^#^	38.0 ± 3.5^b^^,^^c^^,^^#^	36.3 ± 4.0^b^^,^^#^	36.0 ± 4.9^b^^,^^#^
WLD	38.8 ± 5.3^b^^,^^#^	37.5 ± 6.1^b^^,^^#^^,^^$^	33.6 ± 5.8^b^^,^^$^	27.1 ± 6.3^a^^,^^%^	24.0 ± 4.6^a^^,^^%^
IBW	18.7 ± 4.0^a^^,^^#^	19.4 ± 3.0^a^^,^^#^	23.1 ± 2.7^a^^,^^#^^,^^$^	25.3 ± 2.5^a^^,^^$^	23.7 ± 2.2^a^^,^^#^^,^^$^
**Lean mass (kg)**					
ObN	9.0 ± 0.6^a^^,^^b^^,^^#^	8.5 ± 0.7^a^^,^^#^	8.5 ± 0.6^a^^,^^#^	8.4 ± 0.9^a^^,^^#^	8.4 ± 0.7^a^^,^^#^
ObP	10.1 ± 0.9^c^^,^^#^	10.0 ± 1.1^b^^,^^#^	9.8 ± 0.7^b^^,^^#^	9.8 ± 0.8^b^^,^^#^	9.7 ± 0.8^c^^,^^#^
WLD	9.6 ± 0.4^b^^,^^c^^,^^#^	9.2 ± 0.2^a^^,^^b^^,^^#^	9.1 ± 0.2^a^^,^^b^^,^^#^	9.2 ± 0.4^b^^,^^#^	9.1 ± 0.2^b^^,^^c^^,^^#^
IBW	8.6 ± 0.8^a^^,^^#^	8.7 ± 0.6^a^^,^^#^	8.7 ± 0.6^a^^,^^#^	8.6 ± 0.6^a^^,^^#^	8.6 ± 0.5^a^^,^^b^^,^^#^
**Lean (%)**					
ObN	54.4 ± 2.9^a^^,^^#^	54.3 ± 3.0^a^^,^^#^	57.4 ± 2.5^a^^,^^#^^,^^$^	61.1 ± 2.8^a^^,^^$^^,^^%^	65.7 ± 5.1^a^^,^^%^
ObP	58.6 ± 3.4^a^^,^^#^	59.7 ± 3.5^a^^,^^#^	59.1 ± 3.4^a^^,^^b^^,^^#^	60.7 ± 4.0^a^^,^^#^	60.9 ± 4.9^a^^,^^#^
WLD	58.5 ± 5.0^a^^,^^#^	59.6 ± 5.8^a^^,^^#^	63.3 ± 5.6^b^^,^^#^^,^^$^	69.2 ± 5.8^b^^,^^$^^,^^%^	72.6 ± 4.6^b^^,^^%^
IBW	77.6 ± 4.0^b^^,^^#^	76.9 ± 2.9^b^^,^^#^	73.4 ± 2.8^c^^,^^#^^,^^$^	71.3 ± 2.5^b^^,^^$^	72.7 ± 2.3^b^^,^^#^^,^^$^
**DEXA total mass (kg)**					
ObN	16.5 ± 0.6^b^^,^^#^	15.7 ± 0.7^b^^,^^#^^,^^$^	14.9 ± 1.0^b^^,^^$^^,^^%^	13.7 ± 1.4^b^^,^^%^^,^^∧^	12.8 ± 1.4^b^^,^^∧^
ObP	17.2 ± 1.5^b^^,^^#^	16.8 ± 1.2^b^^,^^#^	16.6 ± 0.8^c^^,^^#^	16.1 ± 0.6^c^^,^^#^	15.9 ± 0.5^c^^,^^#^
WLD	16.5 ± 1.5^b^^,^^#^	15.6 ± 1.4^b^^,^^#^^,^^$^	14.5 ± 1.0^b^^,^^$^^,^^%^	13.3 ± 0.8^b^^,^^%^^,^^∧^	12.6 ± 1.0^a^^,^^b^^,^^∧^
IBW	11.1 ± 0.8^a^^,^^#^	11.3 ± 0.7^a^^,^^#^	11.9 ± 0.7^a^^,^^#^	12.0 ± 0.7^a^^,^^#^	11.8 ± 0.7^a^^,^^#^

*Numerical data presented as mean ± standard deviation*.

^*^ObN, obese nutraceutical group; ObP, obese placebo group; WLD, weight loss diet group; IBW, ideal body weight group.

*Except where otherwise indicated, n = 5 for obesification groups (ObN, ObP, WLD) and n = 6 for group IBW*.

1 *WLD in this column determined from 4 dogs, because 1 dog reached ideal weight and ended treatment at 8 weeks*.

a,b,c *Within a column, values with different superscript letters differ significantly (p < 0.05)*.

#,$,%,∧ *Across columns, values with different superscript symbols differ significantly (p < 0.05)*.

#### Fat Mass

Within and across obesification groups, both absolute (AF) and percent fat mass (%BF) did not significantly differ at treatment baseline or 1 week into treatment, although ObN exhibited higher baseline mean %BF (42.9%) than ObP (38.7%) or WLD (38.8%). Within groups, ObN and WLD lost AF and %BF compared to baseline (*p* < 0.0001), while ObP did not. At study termination, WLD AF was significantly less than ObN (*p* = 0.0173) and ObP (*p* < 0.0001). ObN AF was also less than ObP (*p* < 0.0001). %BF of WLD was lower than ObN and ObP (*p* < 0.0001) at 12 weeks (WLD, 24%; ObN, 30.8%; ObP, 36.0%), but ObN and ObP did not significantly differ from each other. However, change from baseline show that ObN and WLD lost similar levels of AF and %BF after 12 weeks of treatment and more than ObP (*p* < 0.0001; Table 4). Based on baseline vs. end of study comparisons, including the WLD dog that ended study participation at 8 weeks, WLD end of study AF did not differ from ObN, but both were significantly less than ObP (ObN, 4.0 ± 1.0; ObP, 5.7 ± 0.8; WLD, 3.0 ± 0.7; *p* < 0.0001).

#### Lean Mass

Within groups, absolute lean mass (AL) was static across time. Percent lean mass (%LM) was higher at study termination compared to the baseline for ObN and WLD (*p* < 0.0001), not ObP. Baseline AL was higher in ObP vs. ObN (*p* = 0.0064), not vs. WLD. At the end of treatment, AL did not differ between ObP and WLD, but ObN was significantly lower than ObP (*p* < 0.0001) and WLD (*p* = 0.0431). %LM was consistent both within and across obesification groups at baseline and 1 week. By the end of treatment, WLD %LM was higher than ObN and ObP (*p* < 0.0003), which did not differ. Based on change from baseline, all three obesification groups maintained similar levels of AL, but ObN and WLD gained more %LM after 12 weeks of treatment compared to ObP (*p* < 0.0001; Table 4).

### Negative Control (Group IBW)

BW, AF, and %BF increased at 8 weeks compared to baseline (*p* < 0.0086) and decreased back to baseline levels at week 12. BCS, LM, and DEXA total mass were consistent for the duration of the study. %LM decreased over time, although the 12-week level did not differ from baseline. Over time, calories offered (and subsequent caloric intake; *p* < 0.0001) was significantly decreased to maintain ideal BW. Compared to baseline, calories offered (kcal/BW_kg_^0.75^) was decreased by ~35% at study termination in order to maintain ideal BW. This end of study value (139.8 ± 38.9 kcal/BW_kg_^0.75^) did not differ from ObP (134.1 ± 17.3 kcal/BW_kg_^0.75^) or WLD (117.5 ± 31.1 kcal/BW_kg_^0.75^), but was greater than ObN (101.3 ± 12.7 kcal/BW_kg_^0.75^; *p* = 0.0004).

## Discussion

These data show overweight dogs consuming maintenance energy levels for their ideal weight achieve similar weight loss results when fed a canned adult diet with a leucine/pyridoxine nutraceutical or a canned therapeutic weight loss diet. This is evident in the significantly reduced body weight after 12 weeks of treatment, which did not differ between the two groups. Nutraceutical dogs lost 3.6 ± 0.9 kg body weight and 3.1 ± 0.6 kg fat mass compared to 4.4 ± 1.1 kg body weight and 3.9 ± 0.8 kg fat mass in dogs consuming a therapeutic weight loss diet. The proportion of weight lost as fat in both groups was similar, despite greater overall caloric intake in the WLD group compared to ObN, with nutraceutical dogs losing 86.1% as fat vs. 88.6% in weight loss diet dogs. These results echo human data in which the nutraceutical blend, in a dose modified for people (2.25 mg leucine and 30 mg pyridoxine), was associated with a total fat mass loss of 1.82 ± 0.70 kg vs. no change of fat mass in the placebo group when consuming a maintenance amount of calories. When calorically restricted (500 kcal/day reduction), nutraceutical human subjects lost significantly more total weight (8.15 ± 1.33 vs. 5.25 ± 1.13 kg, *p* < 0.01) and fat mass (7.00 ± 0.95 vs. 4.22 ± 0.74 kg, *p* < 0.01) than the placebo group over the 6-month time frame (21).

Leucine modulates fat oxidation and energy partitioning between adipose tissue and skeletal muscle by diminishing adipocyte lipid storage, increasing net fat oxidation, and decreasing overall adiposity (21, 23–28). This effect is via activation of the mitochondrial biogenesis gene sirtuin1 (SIRT1). SIRT1 is activated by high nicotinamide adenine dinucleotide (NAD+) levels seen during times of high energy demand (e.g., caloric restriction, fasting, and exercise), leading to a general switch from a glycolytic state to one of oxidative phosphorylation (33, 34). Leucine serves as an allosteric activator of SIRT1, reducing the Km for NAD^+^ and thereby facilitating SIRT1 activation at the lower NAD^+^ concentrations that characterize the metabolically replete state (35). To achieve this effect, leucine needs to be increased from normal fasting levels (~0.1 mM) to plasma levels of ~0.4–0.5 mM (27, 36, 37). Providing leucine in a single bolus form results in plasma levels of ~0.5 mM vs. a plasma leucine response to protein-rich meals of ~0.25 mM. Thus, although total leucine intake was similar across groups in the present study, the timing of leucine administration (bolus administration) nonetheless results in a greater plasma concentration necessary for SIRT1 activation. While muscle SIRT1 tissue expression initially increased in ObN dogs, it normalized by study completion and SIRT1 adipose tissue expression did not differ by group or time point, likely due to the similar leucine intake across groups [(38). Effect of a Leucine/Pyridoxine Nutraceutical on Energy Metabolism and Satiety in Lean and Obese Dogs [unpublished doctoral dissertation]. [Knoxville (TN)]: University of Tennessee].

The active form of pyridoxine, pyridoxal 5′ phosphate (PLP), has the ability to attenuate calcium signaling, inhibiting calcium movement into adipocytes *in vitro* and subsequent adipocyte fatty acid synthase (FASN) expression (20, 39–41). In adult dogs, the recommended leucine allowance is 1.7 g/1000 kcal (0.22 g/BW_kg_^0.75^), and the recommended pyridoxine allowance is 0.375 mg/1,000 kcal (0.049 mg/BW_kg_^0.75^) (42). Based on an average weight of 13.82 kg and 864 kcal/day consumption for the 15 dogs from groups ObN, ObP, and WLD used in final data analysis, they should have been receiving at least 1.5 g leucine and 0.32 mg pyridoxine with diet or diet and treatment per day to meet the allowances. All groups were 65.3–71.7% above the allowance for leucine and 84.8–97.9% above the recommended allowance for pyridoxine. Leucine intake was similar across the groups, but ObN was receiving 5–7x more pyridoxine vs. ObP and WLD, potentially indicating this nutrient as the driving force for the supplement's effect.

The ability to induce weight loss via nutraceutical supplementation without extreme energy restriction is an exciting prospect in dogs since it remains the main method for weight loss. Multiple recommendations regarding restriction levels have been published, including 0.5–0.75 × MER_ideal_ (13, 14, 43), 50–100 kcal/BW_kg(ideal)_^0.75^ (44, 45), 0.6 × MER of a weight 15% below current weight (46), or 0.45–0.55 × [95 × kcal/BW_kg(ideal)_^0.75^] (47). It is generally recommended for dogs to maintain a weight loss rate of 1–2%/week on a restricted intake weight loss plan and dogs in the current study averaged 1.8% (nutraceutical), 2.2% (weight loss diet), and 0.5% (placebo) weekly weight loss while consuming a maintenance level of calories for ideal weight over 12 weeks. While ObP dogs did lose weight, this loss was not significant over time and is likely attributable to the initial adjustment to removal of obesification excess calories.

A major strength of the current study is the use of both positive and negative controls. The leucine/pyridoxine nutraceutical was directly compared to a group of dogs under the same feeding conditions while being supplemented with a daily placebo, which is considered the most robust form of clinical trials (48). An active comparator group in the form of a therapeutic WLD also allowed for efficacy comparison of the leucine/pyridoxine nutraceutical to a standard form of obesity treatment. While blinding would have been ideal, the texture difference between the contents of the leucine/pyridoxine nutraceutical vs. the cornstarch placebo and between the CAD vs. the high fiber WLD made maintaining observer blinding difficult. Finally, a group of dogs that did not undergo any specific supplementation and were fed to maintain an ideal weight for the duration of the study were included as a no treatment control. These control groups, along with the use of laboratory-housed animals in which feeding and medicating are regulated by research staff, improve the reliability of the current results and avoid the common problem of inconsistent owner adherence to weight loss programs (11, 14–18). Additional investigation of the leucine/pyridoxine nutraceutical in naturally obese client-owned dogs is now warranted based on these results.

A potential critique of the current study is that the data are based on a low number of dogs. Based on power calculations, six dogs per group were considered sufficient to demonstrate significant differences. Each group started with six dogs, but three dogs (one from each obesification group) did not reach the required 30% BF threshold during obesification and were dropped from analysis. It is possible that having at least six dogs reach the end of the study in each group would have resulted in a different analysis outcome. For this reason, this data should be considered a pilot foundation for additional research in naturally obese client-owned dogs.

Another potential critique is that nutraceutical dogs were being offered significantly less calories than placebo or weight loss diet dogs for the duration of the study, which may have contributed to the perceived effect. However, calories consumed based on metabolic body weight (vs. calories offered) was not different between the placebo and nutraceutical group at the conclusion of the intervention phase. It should be noted that the initial weight changes in response to feeding during the pre-study period suggests a naturally lower MER in the nutraceutical group. When compared against the lowest reported overall MER of dogs (54.5 kcal/kg^0.75^; ~0.6 × MER_ideal_), only one of the ObN dogs was being fed below this level by the end of obesification (49). Dropping this dog from analysis as an outlier was considered, but evidence supporting the existence of a naturally lower MER, rather than simple underfeeding, was considered. This dog achieved both high absolute (6.5 kg) and percent (41.8%) body fat levels by the end of obesification, despite being offered a relatively low energy level from CAD. During obesification, all dogs were fed the same level of oil supplementation, and this dog was being offered less kitten food than 13 of the 15 obesification dogs used in the final analysis, suggesting the excess calorie supplementation does not fully account for his efficient weight gain. It also would have been appropriate to consider re-randomization based on individual energy consumption after MER_ideal_ had been established, but a behavior observation component of the project restricted animal movement in and out of rooms once initial assignments were made.

An additional objective measure of energy requirements, such as calorimetry, would help confirm or refute feeding amounts. Even though predictive equations are considered reliable, predicted resting energy expenditure (REE) only falls within 20% of the REE measure by indirect calorimetry in 51–57% of dogs (50, 51). Because indirect calorimetry measurements cannot be measured on every client-owned animal undergoing a weight loss plan, the predictive equations are used in practice.

## Conclusion

These data show dogs eating maintenance energy levels for ideal body weight of a canned adult diet with a leucine/pyridoxine nutraceutical can achieve weight loss similar to dogs consuming lean maintenance levels of a therapeutic weight loss diet. Both groups performed better than a placebo control, but all groups lost weight over 12 weeks. This supplement shows promise as an alternative method to inducing effective weight loss in dogs without excessive caloric restriction or changing to a therapeutic weight loss diet and additional research in naturally obese client-owned dogs is warranted.

## Data Availability Statement

The raw data supporting the conclusions of this article will be made available by the authors, without undue reservation.

## Ethics Statement

This animal study was reviewed and approved by the University of Tennessee Institutional Animal Care and Use Committee.

## Author's Note

This manuscript represents a portion of a dissertation submitted by MM to the University of Tennessee College of Veterinary Medicine as partial fulfillment of the requirements for a Doctor of Philosophy degree. Presented in abstract form at the American College of Veterinary Internal Medicine Forum, Seattle, WA, June 2013.

## Author Contributions

MM, JB, MZ, CK, and AW-R designed the study, interpreted the data analysis, and reviewed and edited the manuscript. MM prepared the original draft manuscript. All authors approved the final version.

## Conflict of Interest

MZ was employed by the company NuSirt Sciences, Inc. The remaining authors declare that the research was conducted in the absence of any commercial or financial relationships that could be construed as a potential conflict of interest.

## References

[1] Courcier EA Thomson RM Mellor DJ Yam PS. An epidemiological study of environmental factors associated with canine obesity. J Small Anim Pract. (2010) 51:362–7. 10.1111/j.1748-5827.2010.00933.x 20402841

[2] Mao J Xia Z Chen J Yu J. Prevalence and risk factors for canine obesity surveyed in veterinary practices in Beijing, China. Prev Vet Med. (2013) 112:438–42. 10.1016/j.prevetmed.2013.08.012 24042026

[3] Montoya-Alonso JA Bautista-Castaño I Peña C Suárez L Juste MC Tvarijonaviciute A. Prevalence of canine obesity, obesity-related metabolic dysfunction, and relationship with owner obesity in an obesogenic region of Spain. Front Vet Sci. (2017) 4:59. 10.3389/fvets.2017.00059 28487859 PMC5403824

[4] Association for Pet Obesity Prevention 2017 Pet Obesity Survey Results: U.S. Pet Obesity Steadily Increases, Owners and Veterinarians Share Views on Pet Food. (2017). Available online at: https://petobesityprevention.org/2017/ (accessed April 19, 2018).

[5] German AJ Woods GRT Holden SL Brennan L Burke C. Dangerous trends in pet obesity. Vet Rec. (2018) 182:25. 10.1136/vr.k2 29305476 PMC5806590

[6] Lund EM Armstrong PJ Kirk CA Klausner JS Prevalence and risk factors for obesity in adult dogs from private US veterinary practices. Int J Appl Res Vet Med. (2006) 4:177–86. Available online at: https://jarvm.com/articles/Vol4Iss2/Lund.pdf

[7] Kealy RD Lawler DF Ballam JM Mantz SL Biery DN Greeley EH. Effects of diet restriction on life span and age-related changes in dogs. J Am Vet Med Assoc. (2002) 220:1315–20. 10.2460/javma.2002.220.1315 11991408

[8] German AJ Holden SL Wiseman-Orr ML Reid J Nolan AM Biourge V. Quality of life is reduced in obese dogs but improves after successful weight loss. Vet J. (2012) 192:428–34. 10.1016/j.tvjl.2011.09.015 22075257

[9] Yam PS Butowski CF Chitty JL Naughton G Wiseman-Orr ML Parkin T. Impact of canine overweight and obesity on health-related quality of life. Prev Vet Med. (2016) 127:64–69. 10.1016/j.prevetmed.2016.03.013 27094142

[10] Endenburg N Soontararak S Charoensuk C van Lith HA. Quality of life and owner attitude to dog overweight and obesity in Thailand and the Netherlands. BMC Vet Res. (2018) 14:221. 10.1186/s12917-018-1531-z 29986701 PMC6038310

[11] Gentry SJ Results of the clinical use of a standardized weight-loss program in dogs and cats. J Am Anim Hosp Assoc. (1993) 29:369–75.

[12] Laflamme D Kuhlman G The effect of weight-loss regimen on subsequent weight maintenance in dogs. Nutr Res. (1995) 15:1019–28. 10.1016/0271-5317(95)00063-O

[13] Laflamme DP Kuhlman G Lawler DF. Evaluation of weight loss protocols for dogs. J Am Anim Hosp Assoc. (1997) 33:253–9. 10.5326/15473317-33-3-253 9138236

[14] German AJ Holden SL Bissot T Hackett RM Biourge V. Dietary energy restriction and successful weight loss in obese client-owned dogs. J Vet Intern Med. (2007) 21:1174–80. 10.1111/j.1939-1676.2007.tb01934.x 18196722

[15] German AJ Holden SL Bissot T Morris PJ Biourge V. A high protein high fibre diet improves weight loss in obese dogs. Vet J. (2010) 183:294–7. 10.1016/j.tvjl.2008.12.004 19138868

[16] German AJ Holden SL Morris PJ Biourge V. Long-term follow-up after weight management in obese dogs: the role of diet in preventing regain. Vet J. (2012) 192:65–70. 10.1016/j.tvjl.2011.04.001 21570327

[17] German AJ Titcomb JM Holden SL Queau Y Morris PJ Biourge V. Cohort study of the success of controlled weight loss programs for obese dogs. J Vet Intern Med. (2015) 29:1547–55. 10.1111/jvim.13629 26426704 PMC4895666

[18] Flanagan J Bissot T Hours MA Moreno B Feugier A German AJ. Success of a weight loss plan for overweight dogs: the results of an international weight loss study. PLoS ONE. (2017) 12:e0184199. 10.1371/journal.pone.0184199 28886096 PMC5590893

[19] Fritsch DA Ahle NW Jewell DE Allen TA Brejda J Leventhal PS A high-fiber food improves weight loss compared to a high-protein, high-fat food in pet dogs in a home setting. Int J Appl Res Vet Med. (2010) 8:138–45. Available online at: https://www.jarvm.com/articles/Vol8Iss3/Vol8%20Iss3Fritsch1.pdf

[20] Zemel MB Bruckbauer A. Effects of a leucine and pyridoxine-containing nutraceutical on fat oxidation, and oxidative and inflammatory stress in overweight and obese subjects. Nutrients. (2012) 4:529–41. 10.3390/nu4060529 22822451 PMC3397351

[21] Zemel MB Bruckbauer A. Effects of a leucine and pyridoxine-containing nutraceutical on body weight and composition in obese subjects. Diabetes Metab Syndr Obes. (2013) 6:309–15. 10.2147/DMSO.S49623 24003309 PMC3755702

[22] United States Department of Agriculture Methods and Application of Food Composition Laboratory. Beltsville, MD: Nutrient Search (2020). Available online at: https://www.ars.usda.gov/northeast-area/beltsville-md-bhnrc/beltsville-human-nutrition-research-center/methods-and-application-of-food-composition-laboratory/mafcl-site-pages/sr-legacy-nutrient-search/ (accessed August 10, 2020).

[23] Donato J Jr. Pedrosa RG Cruzat VF Pires ISO Tirapegui J. Effects of leucine supplementation on the body composition and protein status of rats submitted to food restriction. Nutrition. (2006) 22:520–27. 10.1016/j.nut.2005.12.008 16600817

[24] Sun X Zemel MB. Leucine and calcium regulate fat metabolism and energy partitioning in murine adipocytes and muscle cells. Lipids. (2007) 42:297–305. 10.1007/s11745-007-3029-5 17406924

[25] Sun X Zemel MB. Leucine modulation of mitochondrial mass and oxygen consumption in skeletal muscle cells and adipocytes. Nutr Metab. (2009) 6:26. 10.1186/1743-7075-6-26 19500359 PMC2701939

[26] Zhang Y Guo K LeBlanc RE Loh D Schwartz GJ Yu YH. Increasing dietary leucine intake reduces diet-induced obesity and improves glucose and cholesterol metabolism in mice via multimechanisms. Diabetes. (2007) 56:1647–54. 10.2337/db07-0123 17360978

[27] Macotela Y Emanuelli B Bång AM Espinoza DO Boucher J Beebe K. Dietary leucine-an environmental modifier of insulin resistance acting on multiple levels of metabolism. PLoS ONE. (2011) 6:e21187. 10.1371/journal.pone.0021187 21731668 PMC3120846

[28] Jiao J Han SF Zhang W Xu JY Tong X Yin XB. Chronic leucine supplementation improves lipid metabolism in C57BL/6J mice fed with a high-fat/cholesterol diet. Food Nutr Res. (2016) 60:31304. 10.3402/fnr.v60.31304 27616737 PMC5018683

[29] Laflamme D Development and validation of a body condition score system for cats: a clinical tool. Feline Pract. (1997) 25:13–18.

[30] Laflamme DP Development and validation of a body condition score system for dogs. Canine Pract. (1997) 22:10–15.

[31] Thatcher CD Hand MS Remillard RL editors. Small animal clinical nutrition: an iterative process. In: Small Animal Clinical Nutrition. 5th ed Topeka, KS: Mark Morris Institute (2010). p. 3–21.

[32] Nagaoka D Mitsuhashi Y Angell R Bigley KE Bauer JE. Re-induction of obese body weight occurs more rapidly and at lower caloric intake in beagles. J Anim Physiol Anim Nutr. (2010) 94:287–92. 10.1111/j.1439-0396.2008.00908.x 19364373

[33] Rodgers JT Lerin C Haas W Gygi SP Spiegelman BM Puigserver P. Nutrient control of glucose homeostasis through a complex of PGC-1alpha and SIRT1. Nature. (2005) 434:113–18. 10.1038/nature03354 15744310

[34] Chalkiadaki A Guarente L. Sirtuins mediate mammalian metabolic responses to nutrient availability. Nat Rev Endocrinol. (2012) 8:287–96. 10.1038/nrendo.2011.225 22249520

[35] Bruckbauer A Zemel MB. Synergistic effects of polyphenols and methylxanthines with Leucine on AMPK/Sirtuin-mediated metabolism in muscle cells and adipocytes. PLoS ONE. (2014) 9:e89166. 10.1371/journal.pone.0089166 24551237 PMC3925247

[36] Bruckbauer A Zemel MB Thorpe T Akula MR Stuckey AC Osborne D. Synergistic effects of leucine and resveratrol on insulin sensitivity and fat metabolism in adipocytes and mice. Nutr Metab. (2012) 9:77. 10.1186/1743-7075-9-77 22913271 PMC3506499

[37] Li H Xu M Lee J He C Xie Z. Leucine supplementation increases SIRT1 expression and prevents mitochondrial dysfunction and metabolic disorders in high-fat diet-induced obese mice. Am J Physiol Endocrinol Metab. (2012) 303:E1234–44. 10.1152/ajpendo.00198.2012 22967499 PMC3517633

[38] Murphy M Effect of a Leucine/Pyridoxine Nutraceutical on Energy Metabolism and Satiety in Lean and Obese Dogs (unpublished doctoral dissertation). Knoxville, TN, University of Tennessee. (2014).

[39] Lal KJ Sharma SK Dakshinamurti K. Regulation of calcium influx into vascular smooth muscle by vitamin B6. Clin Exp Hypertens. (1993) 15:489–500. 10.3109/10641969309041624 7683949

[40] Dakshinamurti K Lal KJ Ganguly PK. Hypertension, calcium channel and pyridoxine (vitamin B6). Mol Cell Biochem. (1998) 188:137–48. 10.1023/A:1006832810292 9823019

[41] Shi H Moustaid-Moussa N Wilkison WO Zemel MB. Role of the sulfonylurea receptor in regulating human adipocyte metabolism. FASEB J. (1999) 13:1833–8. 10.1096/fasebj.13.13.1833 10506587

[42] National Research Council of the National Academies Vitamins. In: Beitz DC Jonker JS editor. Nutrient Requirements of Dogs and Cats. Washington, DC: National Academy Press (2006). p. 193–245.

[43] Borne AT Wolfsheimer KJ Truett AA Kiene J Wojciechowski T Davenport DJ. Differential metabolic effects of energy restriction in dogs using diets varying in fat and fiber content. Obes Res. (1996) 4:337–45. 10.1002/j.1550-8528.1996.tb00241.x 8822758

[44] Markwell PJ Butterwick RF Wills JM Raiha M. Clinical studies in the management of obesity in dogs and cats. Int J Obes Relat Metab Disord. (1994) 18(Suppl. 1):S39–43. 8087164

[45] Diez M Nguyen P Jeusette I Devois C Istasse L Biourge V. Weight loss in obese dogs: evaluation of a high-protein, low-carbohydrate diet. J Nutr. (2002) 132:1685S−7. 10.1093/jn/132.6.1685S 12042493

[46] Mlacnik E Bockstahler BA Müller M Tetrick MA Nap RC Zentek J. Effects of caloric restriction and a moderate or intense physiotherapy program for treatment of lameness in overweight dogs with osteoarthritis. J Am Vet Med Assoc. (2006) 229:1756–60. 10.2460/javma.229.11.1756 17144822

[47] Wakshlag JJ Struble AM Warren BS Maley M Panasevich MR Cummings KJ. Evaluation of dietary energy intake and physical activity in dogs undergoing a controlled weight-loss program. J Am Vet Med Assoc. (2012) 240:413–19. 10.2460/javma.240.4.413 22309013

[48] Staudacher HM Irving PM Lomer MCE Whelan K. The challenges of control groups, placebos and blinding in clinical trials of dietary interventions. Proc Nutr Soc. (2017) 76:203–12. 10.1017/S0029665117002816 28629483

[49] Bermingham EN Thomas DG Cave NJ Morris PJ Butterwick RF German AJ. Energy requirements of adult dogs: a meta-analysis. PLoS ONE. (2014) 9:e109681. 10.1371/journal.pone.0109681 25313818 PMC4196927

[50] O'Toole E Miller CW Wilson BA Mathews KA Davis C Sears W. Comparison of the standard predictive equation for calculation of resting energy expenditure with indirect calorimetry in hospitalized and healthy dogs. J Am Vet Med Assoc. (2004) 225:58–64. 10.2460/javma.2004.225.58 15239474

[51] National Research Council of the National Academies Energy. In: Beitz DC Jonker JS editor. Nutrient Requirements of Dogs and Cats. Washington, DC: National Academy Press (2006). p. 28–48.

